# Supplementary material for: Bring More Data!—A Good Advice? Removing Separation in Logistic Regression by Increasing Sample Size
Source: Int J Environ Res Public Health. 2019 Nov 22;16(23):4658. doi: 10.3390/ijerph16234658 (PMC6926877; doi:10.3390/ijerph16234658)
Supplement: Supplementary file 1 [file ijerph-16-04658-s001.pdf]

Bring more data! – a good advice? Removing separation in logistic regression by increasing sample size

Hana Šinkovec, Angelika Geroldinger and Georg Heinze

Supplementary Materials:

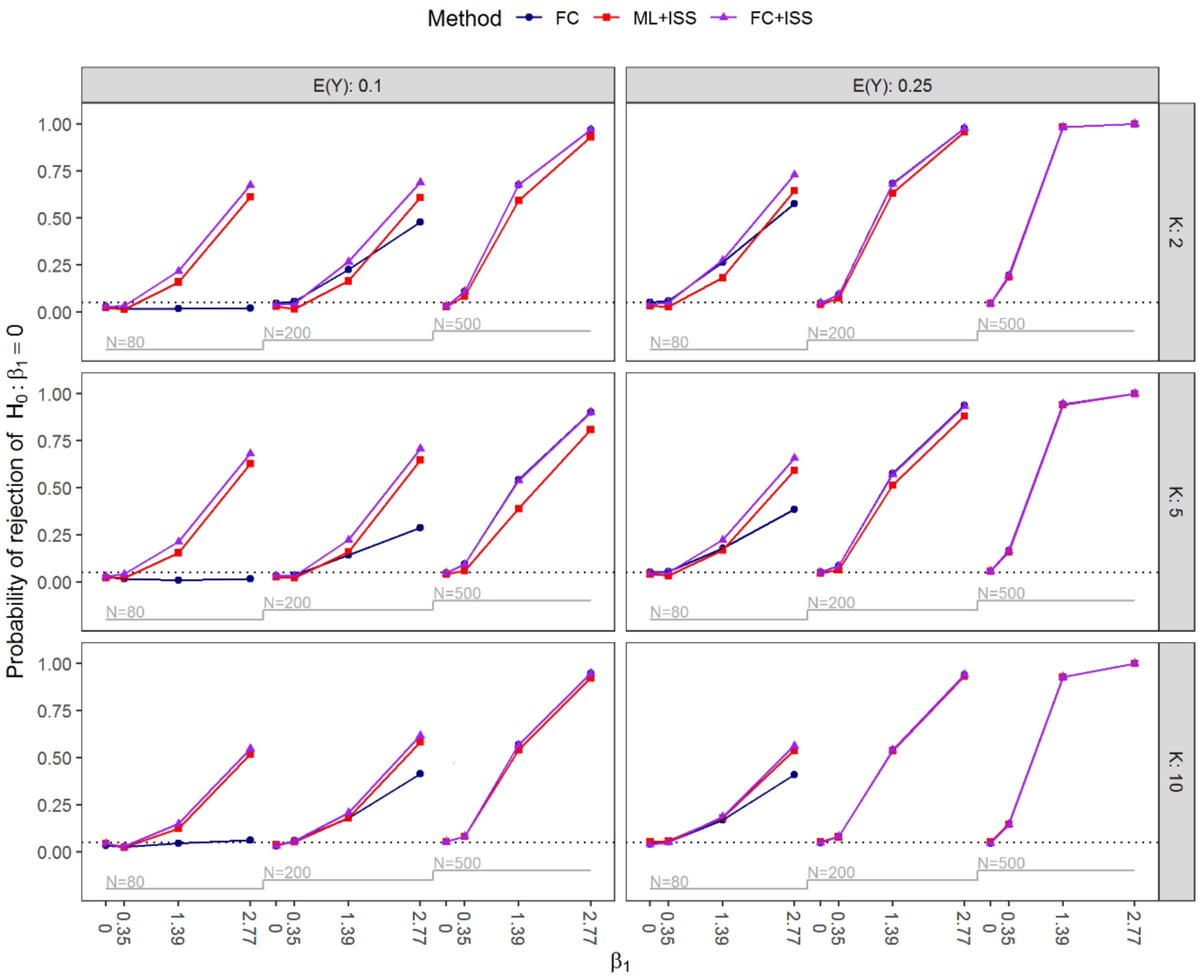

Figure S1. Nested loop plot showing type I error rate ($\beta_1 = 0$) and power ($\beta_1 \neq 0$) for $\hat{\beta}_1$ by the expected value of Y, $\mathrm{E}(Y) \in \{0.1, 0.25\}$, the number of covariates $K \in \{2, 5, 10\}$, the value of $\beta_1 \in \{0, 0.35, 1.39, 2.77\}$ and the sample size $N \in \{80, 200, 500\}$ for all simulated scenarios. FC, Firth's correction; ML+ISS, maximum likelihood combined with the increasing sample size approach; FC+ISS, Firth's correction combined with the increasing sample size approach.

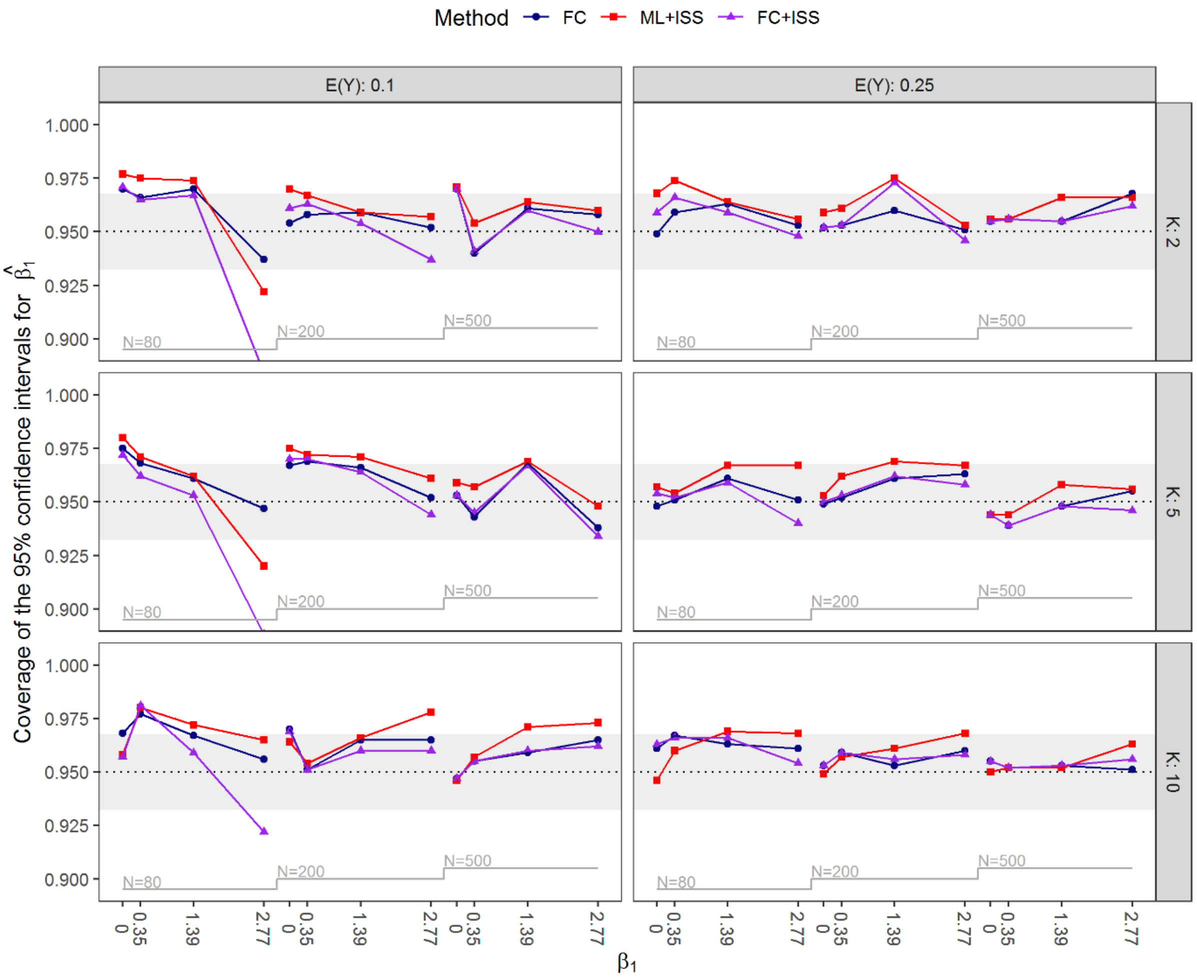

Figure S2. Nested loop plot of coverage of the 95% confidence intervals for $\hat{\beta}_1$ by the expected value of Y, $\mathrm{E}(Y) \in \{0.1, 0.25\}$, the number of covariates $K \in \{2, 5, 10\}$, the value of $\beta_1 \in \{0, 0.35, 1.39, 2.77\}$ and the sample size $N \in \{80, 200, 500\}$ for all simulated scenarios. The grey shaded area marks the 'plausible' interval $0.95 \pm 2.57\sqrt{\frac{0.95 \cdot 0.05}{1000}}$, into which the result of a method with perfect coverage falls with a probability of 0.99. FC, Firth's correction; ML+ISS, maximum likelihood combined with the increasing sample size approach; FC+ISS, Firth's correction combined with the increasing sample size approach.

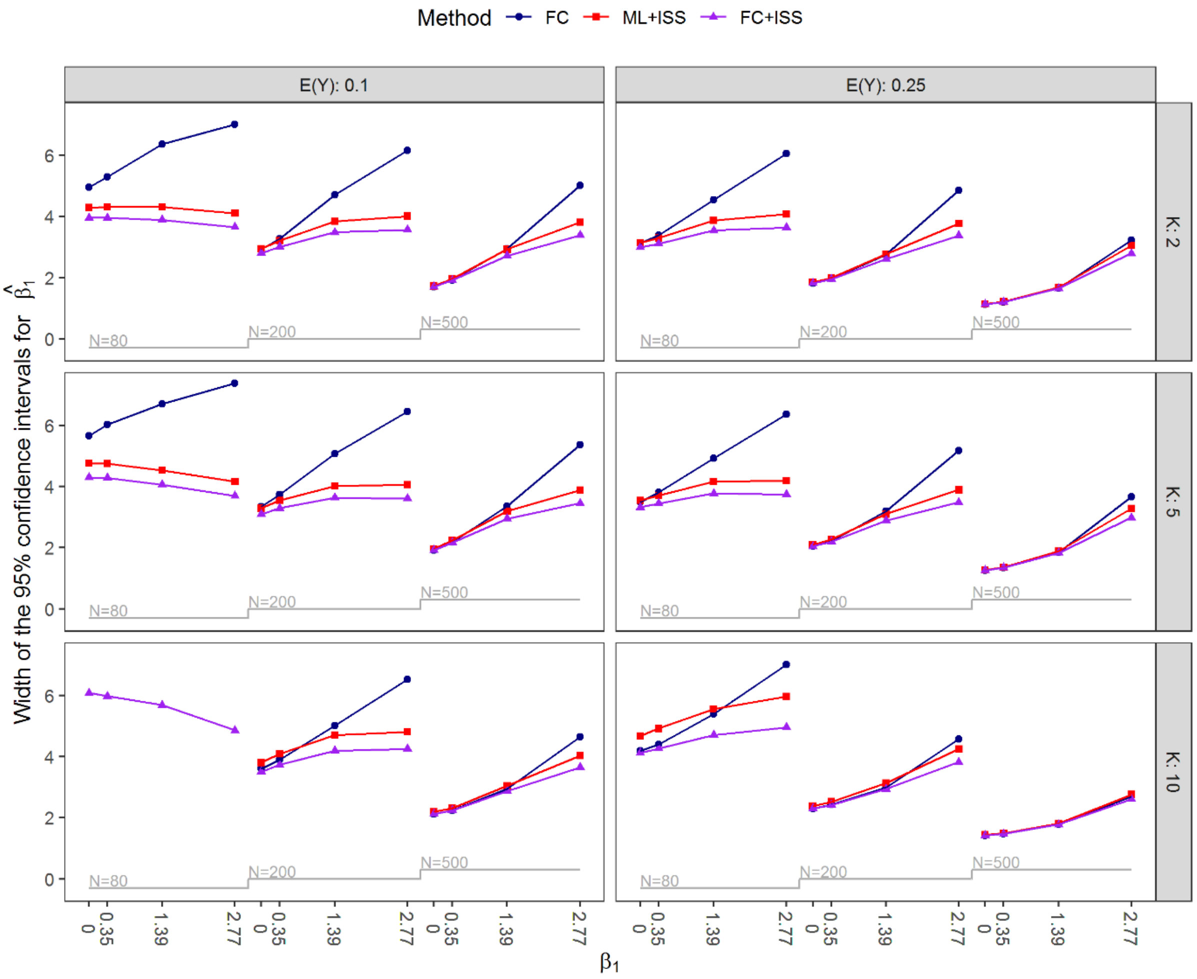

Figure S3. Nested loop plot of width of the 95% confidence intervals for $\hat{\beta}_1$ by the expected value of Y, $\mathrm{E}(Y) \in \{0.1, 0.25\}$, the number of covariates $K \in \{2, 5, 10\}$, the value of $\beta_1 \in \{0, 0.35, 1.39, 2.77\}$ and the sample size $N \in \{80, 200, 500\}$ for all simulated scenarios. The results for FC and ML+ISS for $\mathrm{E}(Y) = 0.1$, $K = 10$ and $N = 80$ are outside of the plot range. FC, Firth's correction; ML+ISS, maximum likelihood combined with the increasing sample size approach; FC+ISS, Firth's correction combined with the increasing sample size approach.
